# Influence of eye tilt on corneal densitometry

**DOI:** 10.1111/opo.13020

**Published:** 2022-06-16

**Authors:** Alejandra Consejo, Marta Jiménez‐García, Jos J. Rozema, Ahmed Abass

**Affiliations:** ^1^ Department of Applied Physics University of Zaragoza Zaragoza Spain; ^2^ Department of Ophthalmology Antwerp University Hospital Edegem Belgium; ^3^ Visual Optics Lab Antwerp (VOLANTIS), Faculty of Medicine and Health Sciences University of Antwerp Antwerp Belgium; ^4^ Department of Mechanical, Materials and Aerospace Engineering, School of Engineering University of Liverpool Liverpool UK; ^5^ Department of Production Engineering and Mechanical Design, Faculty of Engineering Port Said University Port Said Egypt

**Keywords:** cornea, corneal tilt, corneal transparency, densitometry, Pentacam

## Abstract

**Purpose:**

To investigate whether Pentacam densitometry readings are affected by corneal tilt.

**Methods:**

In a prospective study, the right eyes of 86 healthy participants aged 42.8 ± 20.0 years (range 18–79 years) were imaged using Scheimpflug tomography. Elevation maps were exported to calculate corneal tilt using custom‐made software, and densitometry readings were acquired directly from the corneal densitometry analysis add‐on to the standard software Oculus Pentacam HR. Simple mediation analysis was applied to study age as a confounding factor in the correlation between corneal tilt and corneal densitometry.

**Results:**

Corneal tilt and corneal densitometry are not independent from one another because age is significantly correlated with both corneal tilt (*r* = 0.50, *p* < 0.001) and corneal densitometry (*r* = 0.91, *p* < 0.001). Only 3.8% of the correlation between tilt and densitometry operates directly, while the remaining 96.2% depends on age.

**Conclusions:**

Corneal tilt plays a role in corneal densitometry readings, even though the interaction is strongly influenced by age. Age is a well‐known factor in densitometry readings that should be taken into consideration when interpreting Scheimpflug densitometry.

**Key Points**

- Corneal tilt plays a role in corneal densitometry readings, even though that interaction is strongly influenced by age.
- Results suggest strong eye tilt could influence corneal densitometry readings, independent of the origin of the corneal tilt.
- Age is a major confounding factor in corneal densitometry readings that should be taken into consideration when considering a corneal densitometry analysis in a given patient.

## INTRODUCTION

Corneal densitometry measures how much light is backscattered from corneal tissue and can be used as a surrogate for corneal tissue density or corneal transparency.^1^ Different techniques exist to measure corneal transparency, the most popular being the traditional slit‐lamp examination.^2^ Backscatter analysis has demonstrated higher sensitivity in detecting slight transparency changes compared with subjective observation,^3^ and more sophisticated methods, such as spectrophotometry, custom scatterometers, anterior segment–optical coherence tomography (AS‐OCT), confocal microscopy or Scheimpflug imaging,^4^ are needed to quantify changes in corneal transparency objectively.

In the last decade, the Pentacam HR (OCULUS, oculus.de) has become a benchmark in evaluating corneal densitometry thanks to its availability in clinics worldwide.^5^ This provides a powerful tool to investigate both healthy corneas^6^ and eye diseases such as keratoconus,^7^, ^8^, ^9^, ^10^, ^11^, ^12^ Fuchs endothelial dystrophy,^13^ dry eye,^14^ pellucid marginal degeneration,^15^ high myopia^16^ or glaucoma.^17^ Slight hypoxia induced by contact lens wear has been associated with transient increased backscatter.^18^, ^19^, ^20^ An association of corneal densitometry with the disease has been established in multiple myeloma,^21^ Fabry disease^22^ and other rare disorders.^23^, ^24^ Corneal densitometry has been helpful in evaluating corneal integrity after refractive surgery,^25^, ^26^ corneal crosslinking^13^ and trabeculectomy.^27^ Beyond eye disorders and diseases, it has been reported that while corneal densitometry increases with age,^6^, ^28^, ^29^, ^30^ no correlation has been found between corneal keratometry and refractive parameters.^29^

During a Pentacam eye scan, patients are instructed to focus on an internal target. As a result of the mismatch between the optical and visual axes,^31^ topography and tomography maps are tilted systematically.^32^, ^33^, ^34^ Furthermore, the level of eye tilt depends on age^35^ and eye dominancy.^32^

Corneal light scattering, including strong limbal backscatter, is affected by eye orientation relative to the slit‐light source, and consequently, corneal tilt with respect to the visual axis could influence corneal densitometry readings. Consequently, this study aims to investigate whether Pentacam densitometry readings are affected by corneal tilt under natural fixation, measured with a validated, custom algorithm.^33^, ^36^

## METHODS

### Participants

This study was approved by the Antwerp University Hospital Research Ethics Committee and adhered to the tenets of the Declaration of Helsinki. Subjects provided signed informed consent before enrolment. Fully anonymised records from 86 healthy Caucasian subjects (66% women and 34% men) aged between 18 and 78 years, (mean ± SD 42.8 ± 20.0 years) were collected for this prospective study.

All participants underwent a comprehensive ophthalmologic examination, including corneal Scheimpflug imaging using Pentacam. Corneal disease, previous corneal or intraocular surgery, diabetes mellitus, multiple sclerosis or uncontrolled hypertension were considered exclusion criteria, while subjects presenting with exclusively peripheral limbal degenerations associated with ageing such as arcus senilis were included. Only the right eyes were considered in this study to avoid any artefact in the study outcomes as a result of the natural correlation between fellow eyes.^37^

### Estimation of corneal tilt

Raw anterior and posterior corneal height maps were exported for further analysis. A previously validated method^33^, ^36^ was applied to calculate the three‐dimensional angle between the visual and optical axes. This angle (known as angle alpha) was used as a measure of corneal tilt in the current study. The estimation of the visual and optical axes is summarised as follows.

Earlier theoretical analysis and clinical studies demonstrated that eye orientation during a Pentacam examination corresponds to the best approximation of the visual axis.^38^, ^39^ Accordingly, the axis of the Pentacam Scheimpflug camera was considered as the visual axis.

To determine the corneal optical axis, defined as the path of light that goes through the ocular system without refraction,^40^ a raytracing algorithm was custom coded in MATLAB (MathWorks, mathworks.com) and graphically validated using AutoCAD (Autodesk, autodesk.com). In short, the methodology consists of simulating parallel light rays directed towards the cornea and refracted through the anterior and posterior surfaces according to Snell's law.^41^ The angle of incidence was calculated for each ray with respect to the normal line to the anterior and posterior corneal surfaces using ray tracing to provide a measure for the local focal length. The corneal topography of each eye was rotated in three dimensions in an optimisation loop based on the Levenberg–Marquardt nonlinear least‐squares algorithm to maximise the focal length of a central light ray that was selected as the optimal optical axis. The full description of optical axis determination can be found in previous literature.^33^

### Estimation of corneal densitometry

**FIGURE 1:**
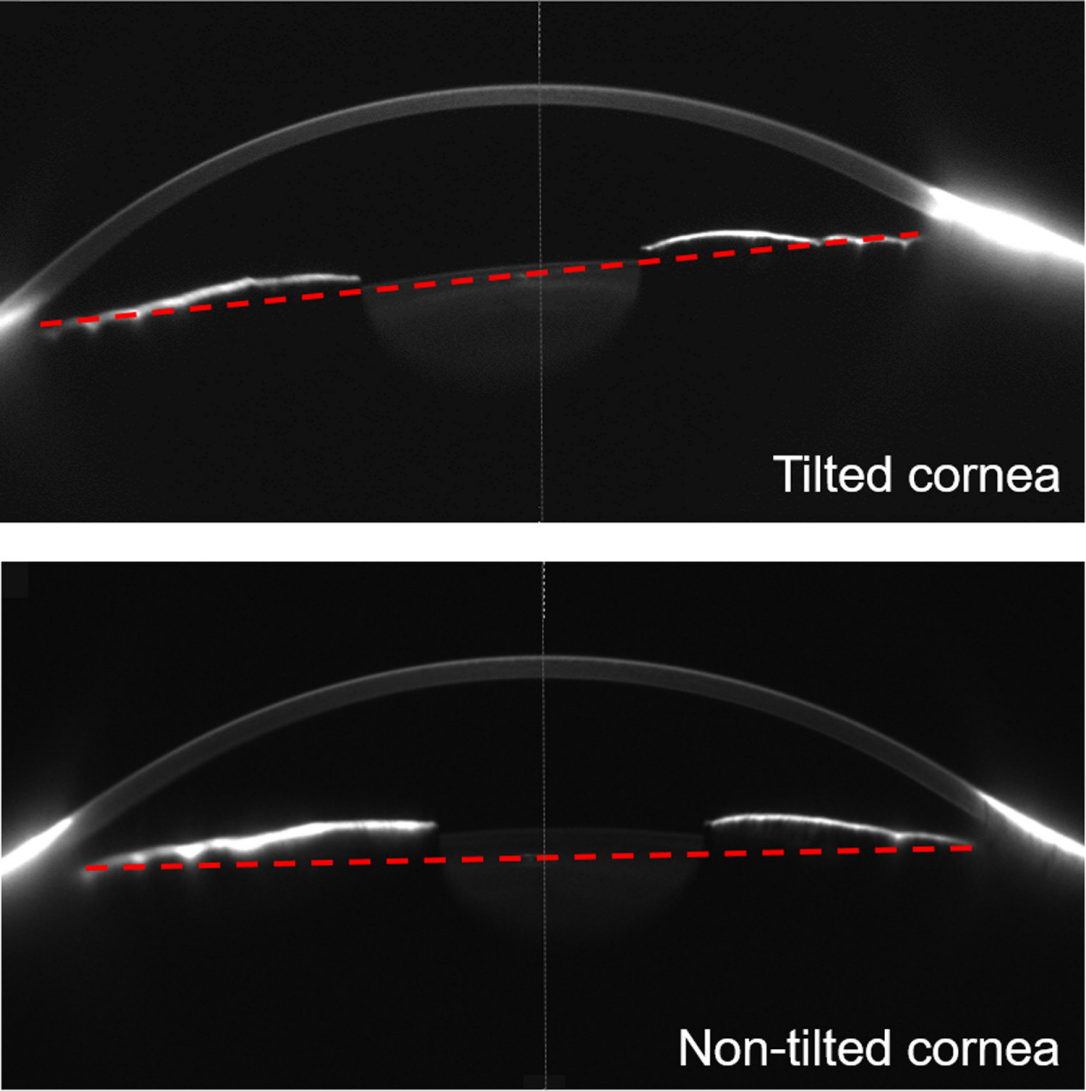
Examples of corneal tomography acquired with the Pentacam. Images correspond to two subjects showing a different level of corneal tilt. The red dashed lines illustrate the level of tilt. The upper image (higher tilt) shows a brighter cornea and stronger limbal reflections than the lower image (smaller tilt).

Figure 1 illustrates two examples of raw corneal imaging with the Pentacam. The corneal densitometry screen is provided as an add‐on to the standard software of the Pentacam Scheimpflug device. The Pentacam measurement protocol takes a series of 25 images over equally distributed meridians. In the postmeasurement processing, data are interpolated to create a densitometry map via the Pentacam software package. The output is expressed in standardised greyscale units (GSU). The standardised greyscale unit measure is calibrated by proprietary software, which defines a minimum light scatter of 0 (maximum transparency) and a maximum light scatter of 100 (minimum transparency). For consistency with the previous literature, the densitometry measurement protocol was performed in a manner described earlier.^6^ This is provided by the Pentacam software in the form of a regional densitometry assessment, with four independent concentric zones: the central zone of 2 mm diameter, and the annuli extending from 2 to 6 mm diameter, 6 to 10 mm diameter and 10 to 12 mm diameter. Therefore, the overall cornea was considered over a diameter of up to 12 mm. Moreover, the software performs a depth analysis over 3 layers: the anterior layer includes the anterior 120 μm, the central layer and the posterior layer, which corresponds to the most posterior 60 μm of the cornea. In addition, the entire corneal depth was also considered.

### Statistical analysis

Statistical analysis was performed using IBM SPSS software for Windows version 25.0 (IBM, ibm.com), supported by the PROCESS 4.0 package (Andrew F Hayes, processmacro.org). The normality of all sets of data was not rejected (Shapiro–Wilk test, *p* > 0.05). Pearson correlation coefficients (*r*) were used to assess relationships within the continuous variables under investigation. Age was considered a confounding factor for corneal tilt and corneal densitometry by means of simple mediation analysis. A simple mediation model is any causal system in which at least one causal antecedent variable X is proposed as influencing an outcome Y through a single intervening variable M.^42^ Two cases were investigated: 1) Corneal tilt (X) influences corneal densitometry (Y) through age as mediator (M) and 2) Age (X) influences corneal densitometry (Y) through corneal tilt as a mediator (M). The level of significance was set to 0.05.

## RESULTS

**FIGURE 2:**
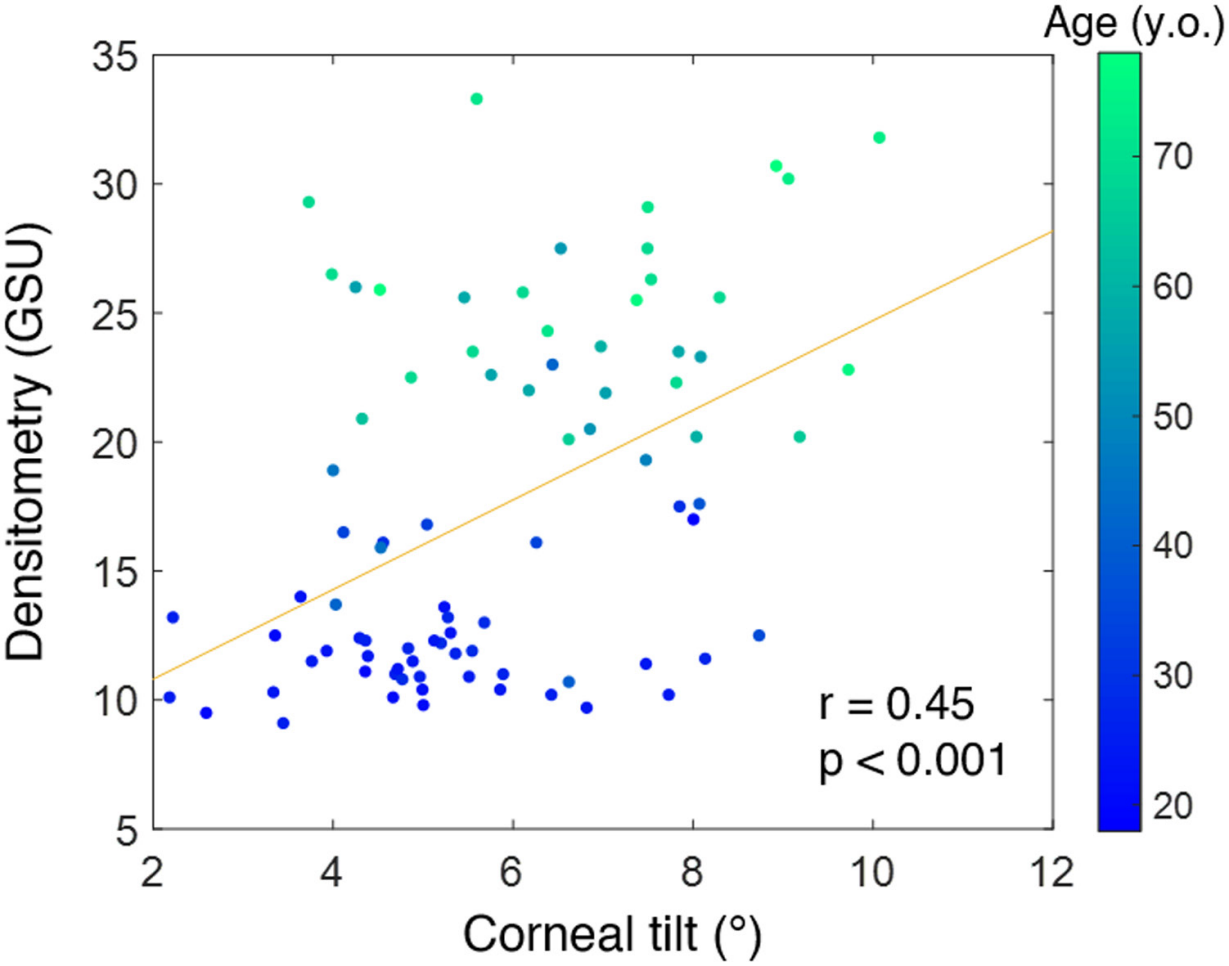
Correlation between corneal densitometry expressed in standardised greyscale units (GSU) and corneal tilt, calculated as the angle between the visual and optical axes. Data points are coloured depending on the age of the subject, as indicated by the colour bar.

When considering corneal tilt and corneal densitometry as independent variables, a significant positive correlation was found between them (*r* = 0.45; *p* < 0.001), as shown in Figure 2. This significant positive correlation is independent of the corneal region or depth, (all, *p* < 0.001), as indicated in Table 1. However, age is significantly correlated with both densitometry (*r* = 0.91, *p* < 0.001) and corneal tilt (*r* = 0.50, *p* < 0.001). Consequently, corneal tilt and corneal densitometry cannot be considered to be independent.

**TABLE 1:** Mean densitometry values ± standard deviation (SD) and range (standardised greyscale units, GSU) for different corneal regions and depths, and the correlation between corneal tilt and densitometry expressed by the Pearson correlation coefficient (*r*).

	Mean ± SD	Range	Densitometry vs tilt (*r*)
Depth layers			
Anterior	23 ± 9	[11, 42]	0.35, *p* < 0.001
Central	16 ± 6	[9, 31]	0.45, *p* < 0.001
Posterior	13 ± 6	[7, 27]	0.43, *p* < 0.001
Concentric regions			
0–2 mm	15 ± 4	[9, 21]	0.48, *p* < 0.001
2–6 mm	14 ± 4	[8, 25]	0.49, *p* < 0.001
6–10 mm	19 ± 9	[8, 45]	0.43, *p* < 0.001
10–12 mm	26 ± 11	[9, 49]	0.41, *p* < 0.001
Overall	17 ± 7	[9, 33]	0.45, *p* < 0.001

Results from the simple mediation analysis (case 1: tilt influences densitometry with age as a mediator) show that only 3.8% of the correlation between tilt and densitometry operates directly, while the remaining 96.2% of that correlation depends on age. The age mediation effect exists and is statistically significant (*p* < 0.05). In other words, corneal tilt on its own does not affect corneal densitometry significantly.

The results from the second simple mediation analysis (case 2: age influences corneal densitometry with corneal tilt as a mediator) show that 91.3% of the correlation between age and densitometry operates directly while the remaining 8.6% of that correlation is dependent on corneal tilt. The tilt mediation effect exists and is statistically significant (*p* < 0.05). Results from case 1 and case 2 simple mediation analyses are consistent. These results show a strong direct effect between age and corneal densitometry, and a minor, but statistically significant, indirect effect of corneal tilt.

The group mean value of corneal tilt was (5.8° ± 1.8°), ranging from 2.1° to 10.0°.

## DISCUSSION

The current study showed that even though corneal densitometry seemed to be affected by corneal tilt (*r* = 0.45; *p* < 0.001), in reality this is an artefact caused by the strong influence of age on both densitometry (*r* = 0.91, *p* < 0.001) and corneal tilt (*r* = 0.50, *p* < 0.001). When considering age as a mediator, the direct correlation between corneal tilt and corneal densitometry greatly weakened. These results highlight the importance of considering age as a confounding factor in densitometry studies. Numerous scientific reports have used corneal densitometry as a tool to investigate eye disease,^7^, ^8^, ^9^, ^10^, ^11^, ^12^, ^13^, ^14^, ^15^, ^16^, ^17^, ^21^, ^22^, ^23^, ^24^, ^25^, ^26^, ^27^ but few consider the potential confounding factors as they seemed to take a statistically significant correlation between two parameters at face value (e.g., tilt and densitometry, Figure 2), when it can in fact be explained entirely by a third variable (age). Clinicians need to be mindful of such confounding factors when using densitometry, or any other clinical test, as a discriminative parameter between groups and consider using mediation analyses where needed.

Unlike other well‐established corneal biomarkers (corneal thickness, curvature, etc.), densitometry does not describe corneal shape but rather corneal tissue properties. To date, there are no other standardised and accessible methods to objectively quantify corneal clarity. Even though the use of densitometry as an eye health marker is still not widespread in clinical practice, many researchers have demonstrated the potential of densitometry as a key diagnostic parameter, for example, in the detection of subclinical keratoconus.^8^, ^12^ Due to its potential and the increasing interest of the community in densitometry, it is of paramount importance to evaluate which potential co‐founding factors could affect it.

A previous study based on bootstrap statistical analysis and an iterative statistical approach concluded that central corneal thickness was not a co‐founding factor in corneal densitometry.^11^ The independence of corneal densitometry and central corneal thickness was also acknowledged elsewhere.^24^ Similarly, no correlations have been found between corneal keratometry and refractive parameters.^29^ To date, age appears to be the strongest confounding factor in densitometry studies. However, further studies should analyse the influence of anterior eye biometry on densitometry readings.

The corneal densitometry values reported here are consistent with those from previous reports.^6^, ^28^, ^29^ Similarly, the group mean value of corneal tilt agrees with that reported by Lopes et al.,^32^ where the mean corneal tilt of the 347 Caucasian participants analysed was 5.9° ± 2.7°.

These results suggest that strong eye tilt could influence corneal densitometry readings, independent of the origin of that tilt. Corneal densitometry is based on the backscattering of light. Generally speaking, light from the source reaches the object to be imaged (the cornea), and is partially backscattered towards the detector to form an image. This final image depends on how light travels within the cornea and how much of it is backscattered.^30^ When an object is tilted from its original position, the light will travel through it in a different manner, and consequently, backscattering will be affected.^43^ Alternative methods to estimate densitometry without using Pentacam software are available for Scheimpflug^43^ and AS‐OCT images.^44^ However, these postprocessing methods do not correct excessive brightness, highlighting the importance of an optimal data acquisition process.

As far as we can tell, the current analysis does not suffer from major issues. We considered an alternative experimental design in which densitometry would have been performed on eyes fixating at different angles. This idea was abandoned, however, in favour of the current approach as this would represent the natural fixation behaviour of the eye.

In conclusion, corneal tilt plays a role in corneal densitometry readings, even though that interaction is strongly influenced by age. Age is a major confounding factor in corneal densitometry readings that should be taken into consideration when considering a corneal densitometry analysis in a given patient.

## AUTHOR CONTRIBUTIONS

**Alejandra Consejo:** Conceptualization (lead); data curation (equal); formal analysis (lead); investigation (equal); methodology (equal); project administration (lead); resources (lead); software (lead); supervision (lead); validation (lead); visualization (lead); writing – original draft (equal); writing – review and editing (equal). **Marta Jiménez‐García:** Data curation (equal); investigation (equal); methodology (equal); writing – original draft (equal); writing – review and editing (equal). **Jos J Rozema:** Data curation (equal); investigation (equal); methodology (equal); resources (lead); writing – original draft (equal); writing – review and editing (equal). **Ahmed Abass:** Conceptualization (lead); data curation (equal); formal analysis (lead); investigation (equal); methodology (lead); project administration (lead); resources (lead); software (lead); supervision (lead); validation (lead); visualization (lead); writing – original draft (equal); writing – review and editing (equal).

## CONFLICTS OF INTEREST

All authors declare that they have no conflicts of interest.
